# A mandatory indication-registration tool in hospital electronic medical records enabling systematic evaluation and benchmarking of the quality of antimicrobial use: a feasibility study

**DOI:** 10.1186/s13756-021-00973-0

**Published:** 2021-07-03

**Authors:** Annemieke K. van den Broek, Berend H. H. Beishuizen, Eric A. F. Haak, Michiel Duyvendak, Jaap ten Oever, Chris Sytsma, Mieke van Triest, Cornelia C. H. Wielders, Jan M. Prins

**Affiliations:** 1 Division of Infectious Diseases, Department of Internal Medicine, Amsterdam UMC, University of Amsterdam, Meibergdreef 9, 1105 AZ Amsterdam, The Netherlands; 2 Center for Infectious Disease Control, National Institute for Public Health and the Environment, Antonie van Leeuwenhoeklaan 9, 3721 MA Bilthoven, The Netherlands; 3 Department of Hospital Pharmacy, OLVG, Location Oost, Oosterpark 9, 1091 AC Amsterdam, The Netherlands; 4 Department of Hospital Pharmacy, Antonius Hospital, Location Sneek, Bolswarderbaan 1, 8601 ZK Sneek, The Netherlands; 5 Department of Internal Medicine, Radboud University Medical Center, Geert Grooteplein Zuid 10, 6525 GA Nijmegen, The Netherlands; 6 Department of Information Technology, Antonius Hospital, Location Sneek, Bolswarderbaan 1, 8601 ZK Sneek, The Netherlands

**Keywords:** Antibiotic prescribing, Antibiotic indication, Antibiotic stewardship, National surveillance, Benchmarking, Quality of care

## Abstract

**Objectives:**

Evaluation of the extent and appropriateness of antimicrobial use is a cornerstone of antibiotic stewardship programs, but it is time-consuming. Documentation of the indication at the moment of prescription might be more time-efficient. We investigated the real-life feasibility of mandatory documentation of the indication for all hospital antibiotic prescriptions for quality evaluation purposes.

**Methods:**

A mandatory prescription-indication format was implemented in the Electronic Medical Record (EMR) of three hospitals using EPIC or ChipSoft HIX software. We evaluated the retrieved data of all antibiotics (J01) prescribed as empiric therapy in adult patients with respiratory tract infections (RTI) or urinary tract infections (UTI), from January through December 2017 in Hospital A, June through October 2019 in Hospital B and May 2019 through June 2020 in Hospital C. Endpoints were the accuracy of the data, defined as agreement between selected indication for the prescription and the documented indication in the EMR, as assessed by manually screening a representative sample of eligible patient records in the EMR of the three hospitals, and appropriateness of the prescriptions, defined as the prescriptions being in accordance with the national guidelines.

**Results:**

The datasets of hospitals A, B and C contained 9588, 338 and 5816 empiric antibiotic prescriptions indicated for RTI or UTI, respectively. The selected indication was in accordance with the documented indication in 96.7% (error rate: 10/300), 78.2% (error rate: 53/243), and 86.9% (error rate: 39/298), respectively. A considerable variation in guideline adherence was seen between the hospitals for severe community acquired pneumonia (adherence rate ranged from 35.4 to 53.0%), complicated UTI (40.0–67.1%) and cystitis (5.6–45.3%).

**Conclusions:**

After local validation of the datasets to verify and optimize accuracy of the data, mandatory documentation of the indication for antibiotics enables a reliable and time-efficient method for systematic registration of the extent and appropriateness of empiric antimicrobial use, which might enable benchmarking both in-hospital and between hospitals.

**Supplementary Information:**

The online version contains supplementary material available at 10.1186/s13756-021-00973-0.

## Background

Antibiotic Stewardship Programmes (ASPs) have been developed to measure and improve the appropriateness of antibiotic use while minimizing unintended consequences of antibiotic use [1–3]. To measure appropriateness, quality indicators (QIs) have been established and validated [4]. One of the QIs is prescribing antimicrobials in accordance with the local guideline, or, if not available, national or international guidelines. Guideline-adherent empiric therapy has shown to be associated with improved clinical outcome [1, 5] .

A frequently used method to evaluate the appropriateness of antimicrobial use in a hospital is the point prevalence survey (PPS), in which all antimicrobial prescriptions and their indications are retrieved during a certain time period [6, 7]. This is done by manually reviewing the (electronic) medical record (EMR). In many cases contact with the attending physician is necessary because of incomplete records, and therefore the evaluation of appropriateness can be very time-consuming. This often results in the evaluation of a relatively small number of patients and a low frequency of analysis, limited to hospitals with available personnel and resources [8]. This calls for a more efficient method to evaluate the appropriateness of antimicrobial use, in order to perform measurements more often or on a larger scale.

EMR tools have already been shown to facilitate ASPs, by computerised decision support, and surveillance of the use of restricted antimicrobials and potential IV-to-oral switch candidates [9–12]. In previous studies it was also shown that in a study setting EMR tools are able to link antibiotic orders to indications, which could facilitate standardized data collection and an automated assessment of antibiotic appropriateness as well [12–18].

The aim of this study was to investigate the real-life feasibility of mandatory documentation of the indication for all antibiotic prescriptions, for the purpose of systematic evaluation of not only the extent, but also the appropriateness of antimicrobial use. This might also facilitate national antimicrobial use surveillance and benchmarking on the hospital level. We implemented a standardized prescription-format in the EMR of three hospitals, two hospitals using EPIC and the other using ChipSoft HIX EMR software, and subsequently extracted the data. This is a feasibility study, as we describe the technical aspects of incorporating these order menus into the EMR and validated the extracted data against the source data in the EMR [19, 20]. In addition, we assessed whether the extracted data can be used to evaluate the compliance rate to national guidelines. For the purpose of this study, we focused on antibiotics prescribed as empiric therapy for patients with respiratory tract infections (RTI) or urinary tract infections (UTI), since these are the most common infections in hospitals.

## Methods

### Study design and setting

The study was performed in three hospitals in the Netherlands. The participating hospitals were the OLVG Hospital (Hospital A), Amsterdam, a 663-bed non-academic teaching hospital, treating more than 500,000 patients annually; the Antonius Hospital (Hospital B), Sneek, a 300-bed non-academic hospital, treating 200,000 patients annually; and the Radboud University Medical Center (Hospital C), Nijmegen, a 593-bed academic teaching hospital, treating over 300,000 patients annually. At all hospitals an ASP is present, including an Antibiotic stewardship team (AST), consisting of an infectious diseases specialist, hospital pharmacist and medical microbiologist. The study consisted of two phases: (1) assessment of feasibility in hospitals using different EMR and prescribing software: Hospital A using EPIC software, and Hospital B using Chipsoft HIX software (August 2018–January 2020); (2) confirmation of feasibility in another hospital using the same EMR and prescribing software: Hospital C using EPIC software (May 2020–September 2020).

Approval from the Institutional review boards was not required for this study because we used retrospective, pseudonymized data for quality optimization purposes. Procedures were in accordance with the General Data Protection Regulation [21].

### Data collection and procedures

A standardized prescription-format was implemented in the EMR and prescribing software of the participating hospitals by software-specific IT specialists. The format obliges physicians to select the indication for the prescription from a predefined list whenever they prescribe an antimicrobial agent to be administered systemically. The possible indications are empiric therapy, targeted therapy or prophylaxis. Subsequently they have to select the main focus of infection, first on tract level, followed by a further specification (Additional file 1: Fig. S1–S3).

Hospital A already implemented the mandatory indication registration in 2015. This prescription format was used as the basis for our feasibility study. Hospital A retrieved data covering the period January 1, 2017, until December 31, 2017. After visual inspection of the data, amendments were made for a more detailed indication registration, which was implemented in the prescription-format of Hospital B in 2018 and Hospital C in 2019. Hospital B provided data covering the period June 1, 2019 until October 31, 2019 and Hospital C covering the period May 14, 2019 until June 9, 2020.

The hospitals extracted datasets from the EMR containing the following parameters:

- Coded patient identifier and admission identifier
- All antibiotic prescriptions for systemic use belonging to Anatomical Therapeutic Chemical (ATC) class J01
- The duration of therapy (start and stop date), dose regimen and route of administration
- The specialty/department of the authorizing and ordering prescriber, and ward of admission of the patient
- Time and date of admission and discharge, i.e. duration of admission
- The chosen focus of infection on tract level, specified in case of RTI or UTI

Further procedures were performed by the authors of the study. For the purpose of this study, we selected the antibiotic prescriptions of all hospitalized patients aged 18 years and older, admitted to any general ward, and receiving empiric antibiotic treatment for an RTI or UTI. Hospitalized clinical patients were defined as patients admitted to the ward for at least 12 h. Empiric therapy was defined as the prescribed antibiotic (combination) therapy at time point 24 h of hospitalization, or the last prescribed antibiotic therapy at the time of discharge in patients who were hospitalized for 12–24 h. This definition of empiric therapy was chosen because febrile patients often receive empiric antibiotic treatment as soon as possible after presentation. During the first hours of admission, incoming diagnostic results may lead to adjustment of the initial indication and therapy. Therefore, we reasoned that the prescriptions that were prescribed at time point 24 h of hospitalization would most accurately reflect the empiric therapy for the indications of interest. After 24 h, empiric therapy is usually adjusted to targeted therapy. We considered antibiotics that were prescribed simultaneously for the same specified indication as antibiotic combination therapy. We excluded ICU patients, because the ICU of Hospital A and B use another EMR; readmissions (defined as an admission within 30 days after the initial hospital discharge), because guideline-recommended empiric treatment is usually not applicable; prescriptions of patients with both RTI and UTI; and erroneous prescriptions, these were prescriptions of which the start date of the antibiotic fell before the date of admission. Furthermore, we excluded the prescriptions for RTI in hospital C that were prescribed after March 2020, because initially no guideline was available for COVID-19 RTI. Exclusion criteria were applied electronically.

The primary endpoint of the study was the accuracy of the dataset, defined as percentage agreement between the selected indication for the prescription and the documented indication in the EMR. The secondary endpoint was the percentage of antibiotic prescriptions in each hospital that was prescribed according to the national guidelines.

### Validation of the dataset

We determined what data had to be extracted from the EMR to be able to select the prescriptions that met the inclusion criteria, and we evaluated the correctness of the datasets. This was first done through general inspection and if deemed necessary through manual chart review of records. Counterintuitive results were resolved. Next, we verified the accuracy of the datasets by manually screening a representative sample of eligible patient records in the EMR of the three hospitals on:

1. whether the indications RTI or UTI and their subsequent specifications selected as indication for the antibiotic prescription were in accordance with the documented diagnosis in the patient record. For this, we screened 200 electronically, randomly selected patient records in Hospital A and C and the 143 patient records with these indications in Hospital B.
2. whether selected indications other than RTI/UTI were in accordance with the documented diagnosis and RTI/UTI infections were thus not accidently excluded. For this we screened 100 electronically, randomly selected records in all hospitals.

### Appropriateness of prescriptions

After validating the dataset, we measured the appropriateness of the prescriptions. This was done by evaluating whether the prescribed antibiotics for the selected indications were in accordance with the national guidelines of the Dutch Working party on Antibiotic Policy (www.swabid.nl), which contain treatment recommendations for all common infections. In the Netherlands, the national guidelines often provide several possible empiric treatment recommendations, from which the local hospital guidelines can select a number of options [22]. By using the national guidelines as a reference, it is possible to benchmark inpatient antibiotic use between hospitals. The prescribed antibiotics were categorised as (A) in accordance with the guideline-recommended first choice agents; (B) in accordance with the guideline-recommended second choice agents; (C) discordant with the guideline. The appropriateness of the prescriptions linked to the RTI/UTI sub-indication “other” was not measured.

### Data analysis

Descriptive data are presented in numbers with or without percentages, for which SAS version 9.4 (SAS Institute Inc., USA) was used. We did not aim to statistically compare the appropriateness of prescriptions between the three hospitals, as the purpose of the study was to show the feasibility of quality measurements with the use of the mandatory prescription-indication tool with subsequent data extraction from the EMR.

## Results

### Dataset characteristics and validation

**Fig. 1:**
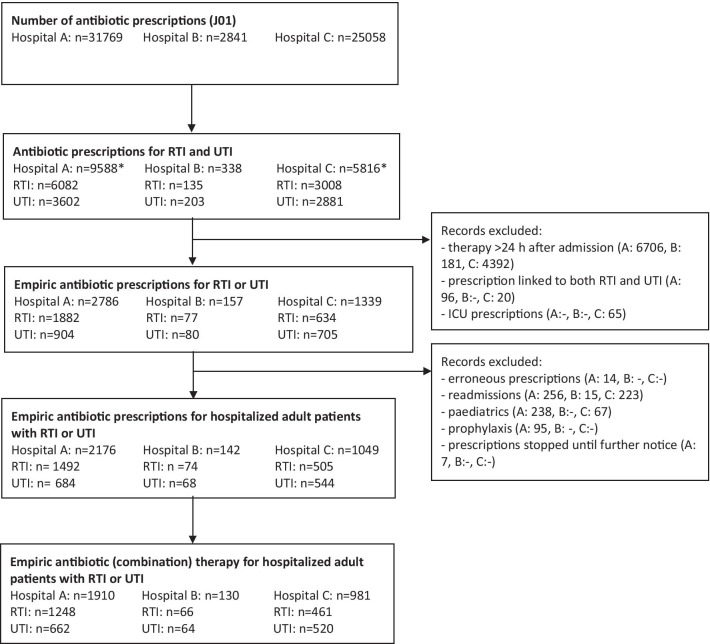
**Antibiotic prescriptions in Hospital A, B and C**. A, Hospital A; B, Hospital B; C, Hospital C. *The number presents the total amount of prescribed antibiotics for RTI and UTI. Some prescriptions were linked to both the indications RTI and UTI. Therefore, the total amount of prescribed antibiotics is lower than the sum of prescriptions indicated for RTI and UTI

The datasets of the three hospitals contained 31,769 (Hospital A), 2841 (Hospital B) and 25,058 (Hospital C) systemic antibiotic (J01) prescriptions, respectively (Fig. 1). Of these, 9588 (30%), 338 (12%) and 5816 (23%), respectively, had the indication RTI or UTI or both.

The datasets were first checked on correctness by general inspection. Counterintuitive results were further investigated and resolved. For example, the number of antibiotic prescriptions in Hospital B was initially very low, which turned out to be caused by the setting of the EMR tool that enabled optional indication registration instead of mandatory registration. For the final dataset we therefore used the data that was extracted after this problem was solved (from June 2019 onwards).

Next, for the data elements provided in addition to the antibiotic prescriptions and indications, we investigated what data needed to be extracted to get the most accurate presentation of our predefined selections. For example, to get the most accurate presentation of the patient’s department of admission, for Hospital A the variable “specialty of authorizing prescriber” had to be extracted, and “specialty of admission” for Hospital B.

Furthermore, we evaluated 87 (n = 30 + 27 + 30 for Hospital A, B and C, respectively) patients in whom hospital-acquired pneumonia (HAP) or prophylaxis was selected. HAP was evaluated to determine what definition for HAP was used by the prescribers in the three hospitals. In all hospitals HAP was (correctly) defined as pneumonia acquired after recent hospital or healthcare centre admission, for instance nursing homes. Prophylaxis was evaluated because the EMR tool in Hospital A required registering the focus of infection when selecting the indication prophylaxis, indicating that the antibiotic might be prescribed as therapy instead of prophylaxis. Evaluation of a sample of the prophylactic prescriptions confirmed that 48 out of 53 (n = 15 + 23 + 15 for Hospital A, B and C, respectively) prescriptions were truly prescribed as prophylaxis and not as therapy, and these prescriptions were therefore justly excluded.

To verify the accuracy of the data, we compared the selected indication with the diagnosis as recorded in the EMR for 300 patients (Hospital A and C) and 243 patients (Hospital B) (Table 1). Overall, the selected indication did not match with the documented diagnosis in 3.3% of the 300 cases in Hospital A, 21.8% of 243 cases in Hospital B and 13.1% of 298 cases in Hospital C. Indication selection errors were mostly due to inaccurate sub-indications. The error rate of Hospital B and C was explained mainly by incorrect selection of cystitis when a complicated UTI (urosepsis or pyelonephritis) was documented in the case notes (n = 37 for Hospital B, and n = 20 for Hospital C). In Hospital C the option “other” was missing as possible RTI-specification, resulting in 7 incorrect selections, as prescribers seemed to select the second best option. Of the randomly selected records with indications other than RTI/UTI, 7 prescriptions indicated for RTI/UTI were missed in Hospital B, only 2 in Hospital C and none in Hospital A. This shows that, depending on the hospital, we have missed a number of prescriptions for RTI/UTI.

**Table 1:** Verification of selected indications

Samples	Hospital A (inaccurate selections/number of screened records)	Hospital B (inaccurate selections/number of screened records)	Hospital C (inaccurate selections/number of screened records)
RTI—error rate (%)	4/100 (4%)	7/70 (10%)	17/99^a^ (17%)
Selected indication versus documented diagnosis	1 prophylaxis ↔ HAP	3 CAP-m ↔ COPD	4 CAP ↔ other
	1 CAP ↔ COPD	1 CAP-s ↔ CAP-m	1 CAP-m ↔ COPD
	1 CAP ↔ bronchitis	2 COPD ↔ CAP-m	1 Bronchitis ↔ COPD
	1 other ↔ skin and soft tissue infections	1 aspiration pneumonia ↔ CAP-s	1 Bronchitis ↔ CAP
			3 CAP ↔ aspiration pneumonia
			3 HAP ↔ other
			1 CAP-s ↔ CAP-m
			2 CAP-m ↔ HAP
			1 CAP-m ↔ prophylaxis
UTI—error rate (%)	6/100 (6%)	39/73 (53%)	20/99^a^ (20%)
Selected indication versus documented diagnosis	5 cystitis ↔ complicated UTI	37 cystitis ↔ complicated UTI	20 cystitis ↔ complicated UTI
	1 prophylaxis ↔ cystitis	1 chronic prostatitis ↔ urosepsis	
		1 other ↔ urosepsis	
Random^b^—error rate (%)	0/100 (0%)	7/100 (7%)	2/100 (2%)
		4 missed UTI	1 missed UTI
		3 missed RTI	1 missed RTI
Total error rate (%)	3.3%	21.8%	13.1%

*CAP-m* community-acquired pneumonia—mild to moderate severe, *CAP-s* community-acquired pneumonia—severe

^a^1 record could not be validated, because documentation regarding the indication of antibiotic treatment was missing/not accessible

^b^Random samples, other than RTI/UTI

### Appropriateness of antibiotic prescriptions

After selecting the empirically prescribed antibiotics and excluding the records that fulfilled exclusion criteria (Fig. 1), 5% of the total amount of prescribed antibiotics remained: 2071 prescriptions for RTI (n = 1492, n = 74 and n = 505, respectively) and 1296 prescriptions for UTI (n = 684, n = 68 and n = 544, respectively).

Prescriptions that were simultaneously prescribed for the same indication were considered combination therapy and were therefore merged for the final analysis of empirically prescribed antibiotic therapy per patient, after which 1775 antibiotic therapies remained for RTI (n = 1248, n = 66 and n = 461) and 1246 for UTI (n = 662, n = 64 and n = 520). The antibiotics prescribed for all RTI and UTI subindications in the three hospitals are presented in Additional file 1: Tables S1–S6.

**Fig. 2:**
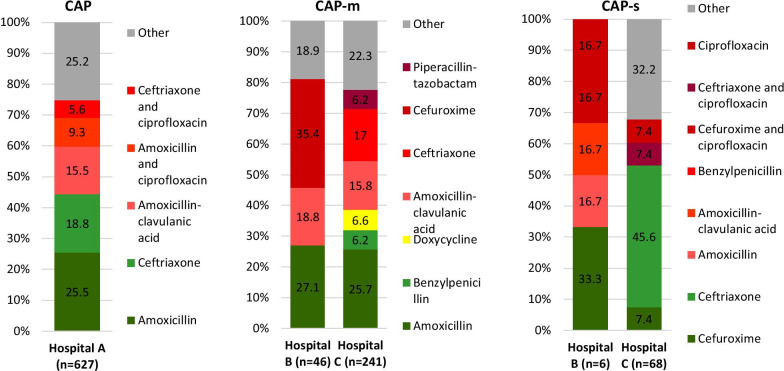
**Appropriateness of antibiotics for RTI**. Category **A** in accordance with the guideline-recommended first choice agents, marked in green; **B** in accordance with the guideline-recommended second choice agents, marked in yellow; **C** discordant with the guideline, marked in red; and other: antibiotics prescribed in less than 5% of cases, marked in grey

**Fig. 3:**
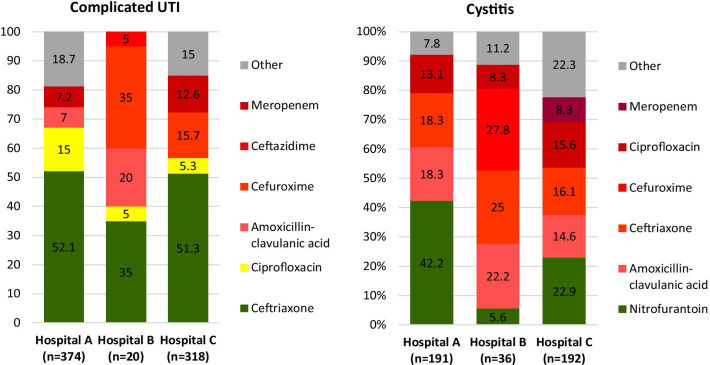
**Appropriateness of antibiotics for UTI**. Category **A** in accordance with the guideline-recommended first choice agents, marked in green; **B** in accordance with the guideline-recommended second choice agents, marked in yellow; **C** discordant with the guideline, marked in red; and other: antibiotics prescribed in less than 5% of cases, marked in grey

The appropriateness of antibiotic therapy for RTI and UTI are presented in Figs. 2 and 3, respectively. The adherence rate to the national guidelines differed considerably between the hospitals, which gives a clear illustration of the opportunities for benchmarking on hospital level.

#### Respiratory tract infections

For Hospital A, mild to moderate–severe and severe CAP were not distinguished in the EMR. For CAP, overall guideline adherence rate was 49.5%, mainly due to the frequent appropriate use of amoxicillin (25.5%) and ceftriaxone (18.8%). Hospital B and Hospital C did distinguish between mild to moderate–severe versus severe CAP, using the CURB-65 score. The adherence rate for mild to moderate–severe CAP was similar for Hospital B and Hospital C (33.4% and 38.5% respectively). The low guideline adherence rate was mainly due to the frequent inappropriate use of cefuroxime in Hospital B (35.4%) and ceftriaxone in Hospital C (17%). In all three hospitals, amoxicillin–clavulanic acid (ACA) was inappropriately prescribed in 15.5–18.8% of cases. The adherence rate for severe CAP was 35.4% for Hospital B and 53.0% for Hospital C. For severe CAP a wide variation of inappropriate therapy combinations was seen.

The guideline adherence rate for COPD exacerbations and HAP (in this study: pneumonia acquired in other health care institutions, for instance nursing homes) are shown in Additional file 1: Fig. S4. The guideline adherence rates ranged from 40.0 to 52.5% for COPD and 51.0–73.2% for HAP.

#### Urinary tract infections

The guideline adherence rate for complicated UTI was 67.1% in Hospital A, 40.0% in Hospital B and 56.6% in Hospital C. In Hospital A and C, complicated UTI was mainly treated with ceftriaxone (51.1% and 51.3% respectively). In Hospital B, complicated UTI was either treated with ceftriaxone, cefuroxime or ACA, of which the last two agents are considered inappropriate. Cefuroxime was the second most prescribed agent in Hospital C as well.

The antibiotic use for cystitis was appropriate in 45.3% in Hospital A, 5.6% in Hospital B and 28.1% in Hospital C. In all hospitals ACA, ceftriaxone or cefuroxime were commonly prescribed, which are all considered inappropriate.

## Discussion

With this study we demonstrated that it is feasible to introduce mandatory documentation of the indication at the moment of antibiotic prescribing, using a prescription format implemented in the EMR. In order to use the data extracted from the EMR for quality measurements, an initial local validation and if indicated optimization of the datasets is necessary, which was shown in our results. The error rate of the prescription-indication tool ranged from 3.3 (Hospital A) to 21.8% (Hospital B)—the latter because cystitis was often not correctly used as indication for the prescription. We also demonstrated that the retrieved data enable the evaluation of the appropriateness of the prescriptions for empiric therapy. For RTI and UTI a considerable variation in guideline adherence was seen between the hospitals, giving an example of the opportunities of benchmarking on hospital level.

In previous studies, the prescription-indication tools were either implemented in hospital-specific software, or the indications were determined by and specific for the institution [16–18, 23, 24]. Therefore, the generalizability of these tools has been subject of debate and quality measurements were restricted to single hospitals [16–18, 23, 24]. In this feasibility study, we implemented the prescription-indication tool in two nationally and internationally widely used EMR software packages (ChipSoft HIX and EPIC). Also, we standardized the indications that the prescribers could choose from, which enabled benchmarking of the results on the national level. More variables can be extracted and data can be further stratified for a more detailed examination of antibiotic use, for example when evaluation of antibiotic use on department/local level is desired. Nevertheless, the output of the extracted data of the EMR may vary between hospitals. For example, we noticed a difference in how the prescriber’s department was displayed and therefore what data had to be extracted to get the most accurate presentation of the prescriber’s department. Little variation in data output is expected for hospitals using ChipSoft HIX software, since ChipSoft HIX has an uniform EMR content. Hospitals using EPIC software, however, are able to personalize the EMR content to the needs of their facility. Thus, before the datasets of other hospitals can be used for comparison, specification of extracted data is necessary.

The feasibility study identified other points of consideration as well. A large number of antibiotic prescriptions were not evaluated for appropriateness. These were mainly the prescriptions that were excluded because they had other indications than RTI/UTI or because they were not considered as empiric therapy. To enable electronic evaluation of the quality of antibiotic use, we focused on empiric therapy because that is prescribed according to general guidelines. The data output did not include patient characteristics or diagnostic results, which precludes electronic evaluation of targeted therapy. In addition, depending on the hospital, a number of prescriptions for RTI and UTI can be missed due to incorrectly selected indications by prescribers. Thus, one should keep in mind that this method enables to measure the appropriateness of a relatively large sample of antibiotic prescription for RTI/UTI and not the appropriateness of all antibiotic prescriptions for that indication.

The rate of discrepancy between selected indication and documented indication in the EMR was comparable to the 74–90% accuracy rates reported by previous studies investigating the validity of automated indication registration [16–18, 23]. In Hospital B and C, mismatches were mainly caused by incorrectly selected cystitis where this should have been complicated UTI (n = 37 and n = 20 respectively). This also explains why cefuroxime and ceftriaxone were the most frequently prescribed agents for cystitis. This underscores that the accuracy rate needs to be considered when using the data for quality measurements and benchmarking. The difference between the accuracy rates of the hospitals might be partially explained by the timing of data extraction: Hospital A implemented the automated tool in 2015, meaning the prescribers had three years to familiarize with the system, while the data from Hospital B and C was extracted only a few months after implementation. Education and feedback to prescribers by local AST may be necessary to increase the accuracy of the data.

Mandatory prescription-indication documentation and the standardized data collection may considerably reduce the workload for local AST [12, 14, 25]. It makes manual data collection for a PPS probably superfluous, as it presents a framework for a more comprehensive approach. The mandatory selection of an indication might be seen as burdensome for prescribers. However, during the evaluation of our feasibility study prescribers informed us that they did not consider the intervention as such. They considered it not labour-intensive or considered it standard patient care. These responses are comparable with what was previously reported by Beardsley and colleagues. In that study prescribers were surveyed on the burden of an automated prescription-indication tool. They judged it to be minor or occasionally burdensome [23]. In the study of ten Oever and colleagues it was shown that the time needed to perform a PPS and report the measurements was 150 and 30 h, respectively [26]. As a result, quality measurements and improvement activities are either performed on smaller scale, or not performed at all. Introducing mandatory indication registration also requires time and expertise. However, as opposed to PPS, the majority of time needs to be invested once, at the start of the project. Thereafter, time is needed to repeat the analysis (semi)annually or quarterly, which is less time consuming than performing a PPS and potentially generates much more data. In our study we already saw that with increasing experience the time needed for the implementation of the mandatory prescription format, data validation and analysis was significantly shorter in Hospital C. This leaves more time for the AST to focus on quality improvement activities. Analysis and benchmarking of the data can be performed by a regional or national party, which also assures independent quality control. Ongoing surveillance of antibiotic use based on yearly results also enables evaluation of the antibiotic use for indications that occur less often, for instance HAP. These are often missed in a PPS or the results are not interpretable because of the small numbers. Finally, benchmarking on the national level facilitates comparison of the appropriateness of antimicrobial use between hospitals, providing additional targets for improvement [14, 27]. This was also demonstrated in our study. We found that ACA was frequently inappropriately prescribed for both CAP as cystitis in all three hospitals. Also, in Hospital B and C, amoxicillin was prescribed in 33.3% of exacerbations COPD. These findings suggest targets not only for local action, but also for national action.

## Limitations

This study is subject to several limitations. First, the accuracy of the datasets relies on accurate indication selection by the prescribers. Human errors are inevitable, and the error rate might fluctuate over time. The accuracy of a random sample of the dataset should therefore be checked regularly, for example yearly. Second, some antibiotics might be prescribed based on pre-admission used antibiotics, previously cultured pathogens, or because of patient allergies. These prescriptions are currently unjustly labelled as being inappropriate. However, in previous studies it was shown that this does not influence the overall guideline adherence rate notable and therefore can be ignored [28]. Another limitation is that the appropriateness of antimicrobial use was defined as being in accordance with the national guidelines, and not the local guidelines. Local guidelines are derived from the national guidelines and may contain adjustments according to local resistance patterns [4]. However, this would mainly pose a difficulty for countries where a wide range of local resistance is observed, which can be solved by benchmarking per region instead of on the national level. In addition, we did not evaluate the appropriateness of treatment duration, which is also an important target to reduce antibiotic consumption [29]. This would require post-discharge antibiotic prescription data, which we could not retrieve from the datasets. And, we did not evaluate the accuracy of provider’s diagnosis, which is another important intervention for ASTs. The evaluation tool in its current form focuses on whether the prescribed antibiotic is in accordance with the guideline for the presumed diagnosis. Finally, we compared three hospitals without considering comparability between these hospitals in terms of type, size and case-mix, which would be preferable for benchmarking on the national level.

## Conclusion

We have demonstrated the real-life feasibility of mandatory documenting the indication of all antibiotics prescribed in EMR using ChipSoft HIX or EPIC software for quality evaluation purposes. It enables a reliable and time-efficient method for systematic registration of the extent and appropriateness of empiric antimicrobial use. Initial local validation and, if necessary, optimization of the datasets, however, is required to assure accuracy of the extracted data. The next step is now to implement this prescription-format in more hospitals in the Netherlands and internationally, for the purpose of national and international benchmarking of the quality of in-hospital antibiotic use. To further improve the quality of prescribing it would also be useful to embed local or national guidelines in the EMR, enabling direct feedback whenever an antibiotic is prescribed.

## Supplementary Information

**Additional file 1. Figures 1-3**. EMR prescription format – Hospital A, B and C.** Tables 1-6**. Antibiotic prescriptions for RTI and UTI in Hospital A, B and C.** Figure 4**. Appropriateness of antibiotics for RTI - COPD and HAP.

## Data Availability

The data that support the findings of this study are available from the participating hospitals but restrictions apply to the availability of these data, which were used under license for the current study, and so are not publicly available. Data are however available from the authors upon reasonable request and with permission of the participating hospitals.

## Abbreviations

ACA: Amoxicillin–clavulanic acid
ASPs: Antibiotic Stewardship Programmes
AST: Antibiotic Stewardship Team
ATC: Anatomical Therapeutic Chemical
CAP-m: Community-acquired pneumonia—mild to moderate severe
CAP-s: Community-acquired pneumonia—severe
EMR: Electronic medical record
HAP: Hospital acquired or healthcare centre associated pneumonia
PPS: Point prevalence survey
QIs: Quality indicators
RTI: Respiratory tract infections
UTI: Urinary tract infections
