# Supplementary material for: A mandatory indication-registration tool in hospital electronic medical records enabling systematic evaluation and benchmarking of the quality of antimicrobial use: a feasibility study
Source: Antimicrob Resist Infect Control. 2021 Jul 3;10:103. doi: 10.1186/s13756-021-00973-0 (PMC8254448; doi:10.1186/s13756-021-00973-0)
Supplement: Supplementary file 1 — Additional file 1. Figures 1-3. EMR prescription format – Hospital A, B and C. Tables 1-6. Antibiotic prescriptions for RTI and UTI in Hospital A, B and C. Figure 4. Appropriateness of antibiotics for RTI - COPD and HAP. [file 13756_2021_973_MOESM1_ESM.docx]

## **Supplementary data**

**Figure 1. EMR prescription format EPIC - Hospital A**

| **1. Select antimicrobial agent** | |
| --- | --- |
| **2. Select indication** | **Empirical therapy**  Other  Prophylaxis  Targeted therapy |
| *In case of prophylaxis: select indication prophylaxis* | Other  SDD  Perioperative |
| **3. Select focus of infection** | Joints and bones  Gastrointestinal  **Kidneys and urinary tract**  **Respiratory tract**  Gynaecology/obstetrics  Intra-abdominal  Tropical diseases  Skin  Cardiovascular  Sepsis of unknown cause  Other  Central Nervous System  Prophylaxis |
| **4. Select specified indication** | |
| ***In case of Respiratory tract*** | **Other**  **Empyema**  **HAP**  **COPD**  **CAP** |
| ***In case of Kidneys and urinary tract*** | **Other**  **CAD infection**  **Complicated urinary tract infection/prostatitis**  **Cystitis** |
| *In case of CNS* | Other  Post-surgery  Brain abscess  Meningitis/encephalitis |
| *In case of Gastrointestinal* | Other  Clostridium  Enteritis/colitis |
| *In case of Joints and bones* | Non-prosthesis infection  Prosthesis infection |
| *In case of Gynecology/obstetrics* | Other  Obstetric infection  STI  PID/TOA |
| *In case of Cardiovascular tract* | PM/ICD  Endocarditis prosthetic valve  Endocarditis native valve |
| *In case ok Skin* | Other  Necrotizing fasciitis  Erysipelas  Cellulitis (incl. wound infections) |
| *In case of Intra-abdominal* | Post-surgery/complication  Biliary tract  SBP/CAPD peritonitis  Perforation  Other  Appendicitis |
| *In case of Tropical diseases* | Other  Malaria |

Abbreviations: SDD= selective digestive tract decontamination; CNS= central nervous system; PID/TOA= pelvic inflammatory disease/tuba-ovarian abscess; PM/ICD= pacemaker/implantable cardioverter-defibrillator; CAP= community-acquired pneumonia; COPD= chronic obstructive pulmonary disease; HAP= hospital acquired or healthcare associated pneumonia; SBP/CAPD= spontaneous bacterial peritonitis/continuous ambulatory peritoneal dialysis

**Figure 2. EMR prescription format Chipsoft – Hospital B**

| **1. Select antimicrobial agent** | |
| --- | --- |
| **2. Select focus of infection** | Gastrointestinal  Prophylaxis  **Kidneys and urinary tract**  **Respiratory tract**  Intra-abdominal  Erysipelas/cellulitis  Joints and bones  Switch  Spondylodiscitis  Prostheses infection  Sepsis of unknown cause  Endocarditis  Meningitis/encephalitis  Other  Genitals  Neutropenic fever |
| **3. Select specified indication** | |
| ***In case of Respiratory tract*** | **Aspiration pneumonia**  **Bronchitis- exacerbation COPD**  **HAP**  **CAP-s**  **CAP-m**  **Other**  **Lung abscess/pleural empyema** |
| ***In case of Kidneys and urinary tract*** | **UTI KTx**  **CAUTI**  **Cystitis**  **KCUTI**  **Urosepsis**  **Pyelonephritis**  **Other**  **Chronic prostatitis** |
| **4. Select indication** | Targeted therapy  **Empirical therapy**  Consulted with microbiologist |

Abbreviations: CAP-m= community-acquired pneumonia – mild to moderate; CAP-s= community-acquired pneumonia severe; HAP= hospital acquired or healthcare associated pneumonia; COPD= chronic obstructive pulmonary disease; CAUTI= catheter-associated urinary tract infection; KCUTI= polycystic kidney urinary tract infection; UTI KTx= urinary tract infection in renal transplant.

**Figure 3. EMR prescription format EPIC - Hospital C**

| **1. Select antimicrobial agent** | |
| --- | --- |
| **2. Select indication** | **Empirical therapy**  Prophylaxis  Targeted therapy |
| **3. Select focus of infection** | Joints and bones  Gastrointestinal  **Kidneys and urinary tract**  **Respiratory tract**  Gynaecology/obstetrics  Eye  Ear-nose-throat  Skin and soft tissue  Cardiovascular  Intra-abdominal  Confirmed bacteraemia/fungaemia  Sepsis of unknown cause  Neutropenic fever  Other  Central Nervous System |
| **4. Select specified indication** | |
| ***In case of Respiratory tract*** | **HAP**  **Aspiration pneumonia**  **CAP: CURB 3-5**  **CAP: CURB 2**  **CAP: CURB 0-1**  **Exacerbation COPD**  **Bronchitis**  **Pleural empyema**  **Lung abscess**  **Fungal pneumonia** |
| ***In case of Kidneys and urinary tract*** | **Chronic prostatitis**  **Pyelonephritis**  **Urosepsis**  **Cystitis** |
| *In case of CNS* | Intracranial epidural abscess  Brain abscess  Myelitis  Encephalitis  Meningitis  Intraspinal subdural empyema  Intraspinal epidural abscess  Intracranial subdural empyema |
| *In case of Gastrointestinal* | Clostridium difficile enterocolitis  Gastritis/enteritis |
| *In case of Joints and bones* | Bacterial arthritis  osteomyelitis  Prosthesis infection |
| *In case of Gynecology/obstetrics* | PID  Endometritis  Cervicitis  Vaginitis  STI |
| *In case of Cardiovascular tract* | Mediastinitis  PM/ICD  Vascular prosthesis infection  Endocarditis  Mycotic aneurysm  Thrombophlebitis  Pericarditis |
| *In case ok Skin and Soft tissue* | Wound infection  Phlebitis  Necrotizing fasciitis  Erysipelas  Cellulitis  Postoperative wound infection |
| *In case of Ear-nose-throat* | Peritonsillar abscess  Otitis media  Otitis externa  Pharyngitis  Acute tonsillitis  Sinusitis  Stomatitis |
| *In case of Eye* | Uveitis  Keratitis  Endophtalmitis  Conjunctivitis  Orbital cellulitis  Blepharitis |
| *In case of Intra-abdominal* | Cholangitis  Cholecystitis  Intra-abdominal abscess  Liver abscess  Peritonitis  Infected necrotizing pancreatitis |

Abbreviations: CNS= central nervous system; PID= pelvic inflammatory diseases; PM/ICD= pacemaker/implantable cardioverter-defibrillator; CAP= community-acquired pneumonia; COPD= chronic obstructive pulmonary disease; HAP= hospital acquired or healthcare associated pneumonia

| Antibiotic treatment | CAP | COPD | HAP | Empyema | Other | Total |
| --- | --- | --- | --- | --- | --- | --- |
| Amoxicillin | 160 (25.4) | 17 (9.5) | 5 (4.6) | 1 (5.6) | 52 (16.5) | 235 (18.8) |
| Amoxicillin and ciprofloxacin | 58 (9.2) | 2 (1.1) | - | - | - | 60 (4.8) |
| Amoxicillin-clavulanic acid | 97 (15.4) | 73 (40.8) | 8 (7.4) | 9 (50) | 107 (33.9) | 294 (23.6) |
| Amoxicillin-clavulanic acid and ciprofloxacin | 16 (2.5) | 7 (3.9) | - | 3 (16.7) | - | 26 (2.1) |
| Azithromycin | - | 2 (1.1) | - | - | 11 (3.5) | 13 (1) |
| Benzylpenicillin | 7 (1.1) | - | - | 1 (5.6) | - | 8 (0.6) |
| Benzylpenicillin and ciprofloxacin | 24 (3.8) | - | - | - | - | 24 (1.9) |
| Benzylpenicillin and metronidazole | - | - | - | 1 (5.6) | - | 1 (0.1) |
| Ceftazidime | 7 (1.1) | 4 (2.2) | 4 (3.7) | - | 8 (2.5) | 23 (1.8) |
| Ceftriaxone | 118 (18.8) | 33 (18.4) | 53 (49.1) | - | 42 (13.3) | 246 (19.7) |
| Ceftriaxone and ciprofloxacin | 35 (5.6) | 6 (3.4) | 10 (9.3) | - | 7 (2.2) | 58 (4.6) |
| Ceftriaxone and metronidazole | - | - | 2 (1.9) | 1 (5.6) | - | 3 (0.2) |
| Cefuroxime | 9 (1.4) | - | - | 1 (5.6) | 7 (2.2) | 17 (1.4) |
| Cefuroxime and ciprofloxacin | 8 (1.3) | - | - | - | - | 8 (0.6) |
| Ciprofloxacin | 26 (4.1) | 7 (3.9) | 6 (5.6) | - | 8 (2.5) | 47 (3.8) |
| Clarithromycin | - | - | - | - | 7 (2.2) | 7 (0.6) |
| Clindamycin | - | - | - | 1 (5.6) | - | 1 (0.1) |
| Cotrimoxazole | - | 3 (1.7) | - | - | - | 3 (0.2) |
| Doxycycline | 17 (2.7) | 19 (10.6) | - | - | 30 (9.5) | 66 (5.3) |
| Meropenem | 9 (1.4) | - | 3 (2.8) | - | 7 (2.2) | 19 (1.5) |
| Other^a^ | 36 (5.7) | 6 (3.4) | 17 (15.7) | - | 30 (9.5) | 89 (7.1) |
| Total | 627 | 179 | 108 | 18 | 316 | 1248 |

**Table 1.** **Antibiotic prescriptions for RTI in Hospital A**

*Numbers are n(%); ^a^Other= antibiotic agents prescribed <1% per indication.*

| Antibiotic treatment | CAP-m^a^ | CAP-s^b^ | COPD | HAP | Aspiration pneumonia | Other | Total |
| --- | --- | --- | --- | --- | --- | --- | --- |
| Amoxicillin | 13 (27.1) | 1 (16.7) | 2 (33.3) |  |  |  | 16 (24.2) |
| Amoxicillin and ciprofloxacin | 1 (2.1) |  |  |  |  |  | 1 (1.5) |
| Amoxicillin-clavulanic acid | 9 (18.8) | 1 (16.7) | 2 (33.3) | 1 (50) | 1 (33.3) | 1 (100) | 15 (22.7) |
| Azithromycin | 2 (4.2) |  | 1 (16.7) |  |  |  | 3 (4.5) |
| Azithromycin and ceftazidime |  |  | 1 (16.7) |  |  |  | 1 (1.5) |
| Ceftriaxone | 1 (2.1) |  |  |  |  |  | 1 (1.5) |
| Cefuroxime | 17 (35.4) | 2 (33.3) |  | 1 (50) | 2 (66.77) |  | 22 (33.3) |
| Cefuroxime and ciprofloxacin | 2 (4.2) | 1 (16.7) |  |  |  |  | 3 (4.5) |
| Ciprofloxacin | 1 (2.1) | 1 (16.7) |  |  |  |  | 2 (3) |
| Doxycycline | 1 (2.1) |  |  |  |  |  | 1 (1.5) |
| Piperacillin-tazobactam | 1 (2.1) |  |  |  |  |  | 1 (1.5) |
| Total | 48 | 6 | 6 | 2 | 3 | 1 | 66 |

**Table 2.** **Antibiotic prescriptions for RTI in Hospital B**

*Numbers are n(%); ^a^CAP-m= CAP mild to moderate severe; ^b^CAP-s= CAP severe*

**Table 3.** **Antibiotic prescriptions for RTI in Hospital C**

| Antibiotic treatment | CAP-m^b^ | CAP-s^c^ | COPD | HAP | Aspiration pneumonia | Empyema  Lung abscess | Other | Total |
| --- | --- | --- | --- | --- | --- | --- | --- | --- |
| Amoxicillin | 62 (25.7) | 2 (2.9) | 5 (33.3) |  |  | 1 (6.7) | 5 (12.8) | 75 (16.3) |
| Amoxicillin-clavulanic acid | 38 (15.8) | 2 (2.9) | 4 (26.7) | 4 (7.1) | 16 (59.3) | 2 (13.3) | 6 (15.4) | 72 (15.6) |
| Azithromycin | 2 (0.8) |  |  |  |  | 4 (26.7) | 4 (10.3) | 10 (2.2) |
| Benzylpenicillin | 15 (6.2) | 1 (1.5) |  |  |  |  |  | 16 (3.5) |
| Ceftazidime | 8 (3.3) |  | 1 (6.7) | 2 (3.6) | 1 (3.7) | 1 (6.7) | 7 (17.9) | 20 (4.3) |
| Ceftriaxone | 41 (17) | 31 (45.6) | 1 (6.7) |  | 1 (3.7) | 3 (20) | 3 (7.7) | 80 (17.4) |
| Ceftriaxone and ciprofloxacin | 3 (1.2) | 5 (7.4) | 1 (6.7) |  | 1 (3.7) |  |  | 10 (2.2) |
| Cefuroxime | 8 (3.3) | 5 (7.4) |  | 1 (1.8) |  |  |  | 14 (3) |
| Ciprofloxacin | 2 (0.8) | 5 (7.4) |  | 1 (1.8) |  |  |  | 8 (1.7) |
| Cotrimoxazole | 4 (1.7) | 2 (2.9) |  | 1 (1.8) |  |  |  | 7 (1.5) |
| Doxycycline | 16 (6.6) | 1 (1.5) | 2 (13.3) |  |  |  | 2 (5.1) | 21 (4.6) |
| Levofloxacin | 6 (2.5) | 1 (1.5) |  |  |  |  | 1 (2.6) | 8 (1.7) |
| Piperacillin-tazobactam | 15 (6.2) | 3 (4.4) |  | 40 (71.4) | 6 (22.2) | 1 (6.7) | 5 (12.8) | 70 (15.2) |
| Other^a^ | 21 (8.7) | 10 (14.7) | 1 (6.7) | 7 (12.5) | 2 (7.4) | 3 (20) | 6 (15.4) | 50 (10.8) |
| Total | 241 | 68 | 15 | 56 | 27 | 15 | 39 | 461 |

*Numbers are n(%) ^a^Other= antibiotic agents prescribed <1% per indication; ^b^CAP-m= CAP mild to moderate severe; ^c^CAP-s= CAP severe*

**Table 4.** **Antibiotic prescriptions for UTI in Hospital A**

| Antibiotic treatment | Complicated UTI | Cystitis | CAUTI^b^ | Other | Total |
| --- | --- | --- | --- | --- | --- |
| Amoxicillin | 11 (2.9) | 4 (2.1) |  | 4 (5.6) | 19 (2.9) |
| Amoxicillin and ciprofloxacin |  |  | 1 (4) |  | 1 (0.2) |
| Amoxicillin-clavulanic acid | 26 (7) | 35 (18.3) | 2 (8) | 3 (4.2) | 66 (10) |
| Amoxicillin-clavulanic acid and gentamicin |  |  | 1 (4) |  | 1 (0.2) |
| Ceftazidime |  |  |  | 1 (1.4) | 1 (0.22) |
| Ceftriaxone | 195 (52.1) | 35 (18.3) | 11 (44) | 25 (34.7) | 266 (40.2) |
| Ceftriaxone and doxycycline |  |  |  | 1 (1.4) | 1 (0.2) |
| Ceftriaxone and gentamicin |  |  | 1 (4) |  | 1 (0.2) |
| Cefuroxime | 18 (4.8) | 2 (1) | 1 (4) | 8 (11.1) | 29 (4.4) |
| Ciprofloxacin | 56 (15) | 25 (13.1) | 4 (16) | 10 (13.9) | 95 (14.4) |
| Clindamycin |  |  |  | 1 (1.4) | 1 (0.2) |
| Cotrimoxazole | 15 (4) |  | 1 (4) | 4 (5.6) | 20 (3) |
| Doxycycline |  |  |  | 2 (2.8) | 2 (0.3) |
| Fosfomycin |  | 4 (2.1) |  |  | 4 (0.6) |
| Gentamicin | 4 (1.1) |  | 1 (4) | 2 (2.8) | 7 (1.1) |
| Meropenem | 27 (7.2) | 3 (1.6) | 1 (4) | 3 (4.2) | 34 (5.1) |
| Metronidazole |  |  |  | 2 (2.8) | 2 (0.3) |
| Nitrofurantoin |  | 81 (42.2) | 1 (4) | 4 (5.6) | 86 (13) |
| Trimethoprim |  | 2 (1) |  | 1 (1.4) | 22 (3.3) |
| Vancomycin |  |  |  | 1 (1.4) | 3 (0.5) |
| Other^a^ | 22 (5.9) | 1 (0.5) |  |  | 1 (0.2) |
| Total | 374 | 191 | 25 | 72 | 662 |

*Numbers are n(%);  ^a^Other= antibiotic agents prescribed <1% per indication; ^b^CAUTI=* *catheter-associated urinary tract infection*

**Table 5: Antibiotic prescriptions for UTI in Hospital B**

| Antibiotic treatment | Complicated UTI | Cystitis | CAUTI^a^ | Other | Total |
| --- | --- | --- | --- | --- | --- |
| Amoxicillin |  | 1 (2.8) |  |  | 1 (1.6) |
| Amoxicillin-clavulanic acid | 4 (20) | 8 (22.2) |  | 1 (33.3) | 13 (20.3) |
| Amoxicillin-clavulanic acid and nitrofurantoin |  | 1 (2.8) |  |  | 1 (1.6) |
| Ceftazidime | 1 (5) |  |  |  | 1 (1.6) |
| Ceftriaxone | 7 (35) | 9 (25) | 1 (20) |  | 17 (26.6) |
| Cefuroxime | 7 (35) | 10 (27.8) | 1 (20) | 1 (33.3) | 19 (29.7) |
| Cefuroxime and ciprofloxacin |  | 1 (2.8) |  |  | 1 (1.6) |
| Cefuroxime and metronidazole |  | 1 (2.8) |  |  | 1 (1.6) |
| Ciprofloxacin | 1 (5) | 3 (8.3) | 1 (20) | 1 (33.3) | 6 (9.4) |
| Flucloxacillin |  |  | 1 (20) |  | 1 (1.6) |
| Meropenem |  |  | 1 (20) |  | 1 (1.6) |
| Nitrofurantoin |  | 2 (5.6) |  |  | 2 (3.1) |
| Total | 20 | 36 | 5 | 3 | 64 |

*Numbers are n(%);  ^a^CAUTI=* *catheter-associated urinary tract infection*

**Table 6: Antibiotic prescriptions for UTI in Hospital C**

| Antibiotic treatment | Complicated UTI | Cystitis | Other | Total |
| --- | --- | --- | --- | --- |
| Amoxicillin | 1 (0.3) | 8 (4.2) |  | 9 (1.7) |
| Amoxicillin-clavulanic acid | 8 (2.5) | 28 (14.6) | 2 (20) | 38 (7.3) |
| Ceftazidime | 13 (4.1) | 4 (2.1) |  | 17 (3.3) |
| Ceftriaxone | 163 (51.3) | 31 (16.1) |  | 194 (37.3) |
| Cefuroxime | 50 (15.7) | 7 (3.6) | 1 (10) | 58 (11.2) |
| Ciprofloxacin | 17 (5.3) | 30 (15.6) | 2 (20) | 49 (9.4) |
| Cotrimoxazole | 3 (0.9) | 7 (3.6) | 1 (10) | 11 (2.1) |
| Meropenem | 40 (12.6) | 16 (8.3) |  | 56 (10.8) |
| Nitrofurantoin | 1 (0.3) | 44 (22.9) | 3 (30) | 48 (9.2) |
| Piperacillin-tazobactam | 10 (3.1) | 2 (1) | 1 (10) | 13 (2.5) |
| Trimethoprim |  | 7 (3.6) |  | 7 (1.3) |
| Other^a^ | 12 (3.8) | 8 (4.2) |  | 20 (2.8) |
| Total | 318 | 192 | 10 | 520 |

*Numbers are n(%);  ^a^Other= antibiotic agents prescribed <1% per indication*

Category A) in accordance with the guideline-recommended first choice agents, marked in green; B) in accordance with the guideline-recommended second choice agents, marked in yellow; C) discordant with the guideline, marked in red; and other: antibiotics prescribed in less than 5% of cases, marked in grey.

**Figure 4: appropriateness of antibiotics for RTI – COPD and HAP**
